# Adherence to COVID-19 preventive measures and its association with intimate partner violence among women in informal settings of Kampala, Uganda

**DOI:** 10.1371/journal.pgph.0000177

**Published:** 2022-04-06

**Authors:** Ronald Anguzu, Allen Kabagenyi, Laura D. Cassidy, Simon Kasasa, Abdul R. Shour, Bernadette N. Musoke, Joan N. Mutyoba

**Affiliations:** 1 Division of Epidemiology, Institute for Health and Equity, Medical College of Wisconsin, Milwaukee, Wisconsin, United States of America; 2 Department of Population Studies, School of Statistics and Planning, College of Business and Management Studies, Makerere University, Kampala, Uganda; 3 Department of Epidemiology and Biostatistics, Makerere University School of Public Health, College of Health Sciences, Kampala, Uganda; 4 Carroll University College of Health Sciences, Waukesha, Wisconsin, United States of America; Tsinghua University, CHINA

## Abstract

Cases of coronavirus disease 2019 (COVID-19) detected, and COVID-19 associated mortality increased since the first case was confirmed in Uganda. While adherence to WHO-recommended measures to disrupt COVID-19 transmission has since been implemented, it has been reported to be sub-optimal. An increase in intimate partner violence (IPV) cases was linked to enforcement of COVID-19 lockdowns and other preventive measures especially in informal settings of Kampala. We determined the association between adherence to COVID-19 preventive measures and intimate partner violence among women dwelling in informal settings in Kampala, Uganda. Between July and October 2020, we conducted a three-month prospective cohort study of 148 women living in informal settlements of Kampala during the COVID-19 lockdown and easing of restrictive measures. Participants were surveyed at baseline, at 3-weeks and 6-weeks (endline). The dependent variable was adherence to COVID-19 preventive measures (remained adherent vs poorly adherent) between baseline and endline surveys. This composite outcome variable was computed from implementing all four variables: social distancing, wearing face masks, frequent hand washing and use of hand sanitizers at baseline and endline surveys. The key independent variable was IPV measured as experiencing at least one form of physical, emotional, or sexual IPV. Covariates were age, education, marital status, household size, occupation, and having problems getting food. Adjusted logistic regression analyses tested the independent association between adherence to COVID-19 preventive measures and intimate partner violence. Among 148 respondents, the mean age (SD) was 32.9 (9.3) years, 58.1% were exposed to at least one form of IPV, and 78.2% had problems getting food. Overall, 10.1% were poorly adherent to COVID-19 preventive measures during the first COVID-19 wave. After controlling for potential confounders, remaining adherent to COVID-19 preventive measures were more likely to experience intimate partner violence when compared to women who were poorly adherent to COVID-19 preventive measures during the first COVID-19 wave in Uganda [OR 3.87 95%CI (1.09, 13.79)]. Proportions of women in informal settlements of Kampala experiencing at least one form of IPV during the first COVID-19 wave is substantial. Remaining adherent to preventive measures for COVID-19 transmission may increase IPV exposure risk among women living in informal settlements in Kampala. Contextualizing COVID-19 interventions to the needs of marginalized and vulnerable women and girls in informal settings of Kampala is warranted. Processes to integrated violence prevention and response strategies into the Uganda COVID-19 prevention strategy are underscored.

## Introduction

Since the first Severe Acute Respiratory syndrome coronavirus 2 (SARS)-CoV2 or coronavirus disease 2019 (COVID-19) cases were confirmed in Wuhan, China in November 2019, 240,940,937 cases of COVID-19 and 4,903,911 COVID-19 related deaths have been confirmed [1]. Relatedly in Uganda, 75,537 COVID-19 cases and 781 COVID-19 related deaths have been confirmed [2] with a case fatality rate of 2.5%. The key non-drug interventions recommended by World Health Organization (WHO) namely social distancing, ‘staying-at-home’ covering one’s mouth when coughing, and sanitizing/washing hands [3] were emphasized. Uganda’s COVID-19 task force engaged in an early proactive responses to the COVID-19 pandemic by proposing and encouraging several measures to disrupt COVID-19 transmission in Uganda [4]. Besides enforcement of movement restrictions to curb community transmissions of COVID-19, other public health measures included screening and testing of symptomatic persons, contact tracing, isolation and quarantine, treatment strategies and risk communication and health promotion. On 21^st^ March 2020, when presidential directives to control community transmission and importation of COVID-19 were enforced, police reports suggested an increase in intimate partner violence (IPV) cases during the COVID-19 lockdown especially among women residing in slum areas [5].

Intimate partner violence which refers to any behavior within an intimate relationship that results in physical, emotional or sexual harm [6] remains a persistent public health problem [7] with 56% of married women experiencing some form of IPV [8]. Either partner can experience violence within an intimate relationship, however, women are disproportionately affected by IPV that is predominantly perpetrated their male intimate partners [9]. Following the first presidential directive in March 2020, cases of household spousal abuse increased during the first week from 46% to 56% [10]. In informal settlements, the burden of IPV is reported to be 7.7% in adolescents who reported IPV victimization [11], 32.2% of youth reported being IPV victims [12] while 44.4% of young girls and women experienced one form of IPV [13].

Subsequent COVID-19 preventive measures such as the lock-downs which are decrees sanctioned by the government to limit people’s movement except for essential reasons [14] became increasingly restrictive [15]. COVID-19 restrictions have also been linked to psychological distress from employment loss which affects livelihoods and quality of life especially in households with high poverty levels trigger IPV with women being disproportionately affected [16]. The COVID-19 lockdown was linked to the high burden of mental health issues [16] and suicidal contemplation among adolescents and young adults in Uganda [17]. Survivors of COVID-19 have also experienced psychosocial challenges such as stigmatization resulting in their isolation and subsequent rejection in some communities [18] including informal settings in urban Uganda.

Adherence to preventive measures remains a central tenet to preventing community transmission of COVID-19 that before the development and global roll-out of COVID-19 vaccines, non-pharmaceutical preventive interventions were strongly recommended by WHO. For example, several studies predicted that after lifting ‘stay at home’ directives, effective implementation of public health responses towards COVID-19 such as consistent and correct face mask use, [19], and effective implementation of social distancing measures [20] could interrupt COVID-19 transmission. However, some unintended effects to at-risk sub-populations such as those under chronic medical care like HIV treatment, urban poor and women were anticipated including gendered effects of IPV on poor healthcare service delivery [21]. Considerable concerns and scepticism grew over what the population-level effects of COVID-19 prevention strategies and policies would have such as increasing violence against women and girls [22]. With prolonged COVID-19 restrictions, adherence to COVID-19 preventive measures could potentially aggravate IPV especially urban poor women living in informal settlements especially with recent reports of community stigmatization towards COVID-19 survivors [23]. Evidence has highlighted how negative socio-cultural, economic effects of specific policies directed to curb COVID-19 preventive transmission has directly impacted households and communities [24]. Some of these include the suspension of public transportation and business operations of that crowd people disproportionately particularly among affected disadvantaged and vulnerable communities such as unemployed, poor women in informal settlements.

Knowledge and awareness of COVID-19 transmission and disease severity is generally high in different Ugandan population [25, 26] despite misconceptions held about the risk of COVID-19 acquisition [27] and recently regarding hesitancy for COVID-19 vaccination [28]. Recent research from Uganda conducted by Lawoko and colleagues showed a high likelihood for COVID-19 risk behaviors among adult refugees with low socio-economic status and those living in rural settlements [29]. Although adherence to COVID-19 prevention measures was reported to be poor during the first phase of the COVID-19 pandemic in Uganda [30] some evidence suggests that women are more adherent to practicing COVID-19 preventive measures when compared to men [25]. A knowledge gap persists on whether and to what extent the adherence to WHO recommended COVID-19 prevention measures influences the occurrence of IPV among women in informal settings in Kampala. The need to establish the extent of adherence to these preventive measures for COVID-19 transmission among vulnerable, urban dwelling women in informal settings of Kampala is warranted in order to inform current national COVID-19 prevention and response efforts. Therefore, our objective was to determine the association between adherence to COVID-19 preventive measures and intimate partner violence among women dwelling in three informal settings of Kampala, Uganda. We hypothesized that IPV exposure would differ among women who remained adherent or were poorly adherent to preventive measures for COVID-19.

## Methods

### Study design, setting and population

This was a three-month, prospective cohort study conducted among women aged 18 years and above, living in informal settings within Kampala district during the periods of national lockdown and easing of restrictions to reduce importation and community transmission of SARS-CoV2 infections. Kampala is the capital city of Uganda, located in the central region of Uganda, in East Africa and is under the administration of Kampala Capital City Authority (KCCA). Nearly half (48.3%) of the urban population in Uganda lives in informal settlements/slums [31]. Kampala district houses one-third (31%) of the urban Ugandan population with a total of fifty-seven informal settlements (slums) located within the five administrative divisions of Kampala namely, Central, Nakawa, Makindye, Lubaga and Kawempe divisions [32].

### Sample size and sampling process

Assuming 22.0% of women experienced physical, sexual or emotional violence by their current/past partner [33] and 80% power, we computed a sample size (N) of 148 women needed to achieve a 95% confidence level and ±5% margin of error. Respondents were recruited from three purposively selected informal settlements in the Kawempe and Makindye divisions of the Kampala district. Using probability-proportionate to size sampling, 50 respondents were recruited from Ki-Mombasa and Kabalagala slums while 48 were recruited from Katanga slum. Within each informal settlement, the first respondent was randomly selected, and subsequent respondents systematically selected from each slum village using the local council (LC) I chairpersons’ register of village residents.

### Data collection

Nine research assistants were trained in data collection to conduct one-on-one interviews with eligible women at the household level. The data collectors were introduced to respondents by local leaders (local council chairpersons or committee members) to respondents’ homes. Pre-tested, structured questionnaires were administered at baseline and two follow-up surveys were conducted after three weeks within 3 months of the COVID-19 lockdown and eased restrictions in Uganda.

## Study measures

### Dependent variable

We computed exposure to intimate partner violence as a composite categorical variable. Items used to collect data on IPV were adopted from the Domestic Violence module contained in the 2016 Uganda Demographic Health Survey as described in detail elsewhere [33, 34]. This study defined current or former ‘partner’ as a participants’ husband or partner in a cohabiting union. We operationalized IPV exposure as having experienced at least one of physical, emotional, and/or sexual forms of IPV based on similar measurements from prior studies conducted in Uganda [35, 36]. Overall IPV exposure variable was computed as follows.

First, physical IPV items with yes/no responses asked participants whether their husband/partner (current or last) in the last two months had ever *‘pushed’*, *‘shook or threw something at them’*, *“slapped them’*, *‘fist punched or hit with something harmful’*, ‘*kicked or dragged’*, *‘strangled or burned’*, *“threatened with a knife/gun or other weapon’* and/or ‘*arm twisted or pulled your hair’*. The final physical IPV variable was coded ‘Yes’ when participants reported experiencing at least physical IPV item and ‘No’ when none of the physical IPV items was experienced in the last two months.

Secondly, emotional IPV items with yes/no responses asked participants whether their husband/partner (current or last) in the last two months had ever ‘*humiliated’*, *‘threatened you with harm’*, *and/or ‘insulted or made you feel bad’*. The final emotional IPV variable was coded ‘Yes’ when participants reported experiencing at least one emotional IPV item and ‘No’ when none of the emotional IPV items was experienced in the last two months.

Thirdly, sexual IPV items with yes/no responses asked participants whether their husband/partner (current or last) in the last two months had ever *‘physically forced her into unwanted sex with him’*, *‘forced her into other unwanted sexual acts’* and/or ‘*physically forced the participant to perform sexual acts when she did not want to’*.

Total IPV in the last two months preceding each survey was generated by combining three items: physical, emotional, and sexual IPV. Women who reported experiencing at least one of physical, emotional, or sexual forms of IPV were coded ‘Yes’ while those who reported no to all three forms of IPV were coded ‘No’.

### Key independent variable

This was adherence to COVID-19 preventive measures in the seven days prior to participating in the study interview. We generated a composite count variable using the following four variables: social or physical distancing (maintaining at least 2 meters from individuals you do not live with), wearing face masks when going outside your home, frequent hand washing (after contact with surfaces or persons) and use of hand sanitizers regularly during the day. These four categorical items had yes/no responses. At the time this study was conducted, WHO recommended these four measures as being the most effective in prevention of COVID-19 transmission [37]. Currently, COVID-19 vaccines proven to be effective in reducing disease severity, hospitalizations and COVID-19 variants [38] are available with emergency use authorization from the US Food and Drug Administration [39]. These four COVID-19 preventive measures were considered feasible for use in Uganda by the Ministry of Health, and particularly essential in the context of informal settings in Kampala during this lockdown and restrictive easing period.

We summed up these four variables on COVID-19 preventive measures to create a scale between 0 to 3 where; (i) “0” represented remaining non-adherent to all four COVID-19 preventive measures at baseline and endline, (ii) “1” represented a shift from adherence to all four preventive measures at baseline to non-adherence of all four preventive measures at endline, (iii) “2” represented a shift from non-adherence to all four preventive measures at baseline to adherence to all four COVID-19 preventive measures at endline and (iv) “3” represented remaining adherent to all four COVID-19 preventive measures at baseline and endline surveys. However, due to low frequencies (less than five) in two sub-categories namely, shifting from non-adherence to adherence and also remaining non-adherent, our regression models omitted these two sub-categories.

We adopted a pragmatic approach to recode adherence to COVID-19 preventive measure variable into a binary categorical variable that reflected changes in adherence to COVID-19 preventive measures over time. Therefore, our overall measure of adherence to COVID-19 preventive measures was categorized into; (i) remained adherent and (ii) poorly adherent to COVID-19 preventive measures between baseline and endline surveys. Remaining adherent was defined as an individual adopting all four COVID-19 preventive measures at baseline and endline. Poorly adherent was operationalized as an individual; (i) remaining non-adherent, (ii) shifting from adherence to non-adherence (iii) shifting from non-adherent to adherent to all four preventive measures from baseline to endline.

### Covariates

All independent variables assessed were categorical. These included the respondent’s age (18–24, 25–34, 35–44, 45–64 years), level of formal education attained (none, primary and secondary or above), marital status (single, married/cohabiting, divorced/widowed), household size (one, two to four and five or more), occupation (employed/self-employed, unemployed/student), and having problems getting food (no/yes).

### Statistical analysis

Categorical variables at baseline were described using frequencies and their respective percentages. At bivariate analysis, unadjusted logistic regression models were run for each variable with the study outcome (intimate partner violence). Adjusted logistic regression analyses were run to test the independent association between adherence to all four COVID-19 preventive measures and intimate partner violence. We accounted for cluster level variance of informal settlements using variance cluster estimators in each logistic regression model. Data were analyzed using Stata/SE v15.1 and statistical significance considered at p-values less than 0.05.

## Results

### Participant characteristics at baseline

We recruited 148 participants at baseline and the response rates were 93.2% and 93.9% at three weeks (midline) and six weeks (endline) respectively (Table 1). Baseline characteristics of participants were; mean (SD) age of 32.9 (9.3) years, 80 (54.1%) had primary-level education, 75 (50.7%) were married/cohabiting, 81 (54.7%) lived in household sizes of 2–4, and 107 (72.3%) were employed/self-employed while 115 (78.2%) reported experiencing difficulty getting food during the COVID-pandemic lockdown period. Regarding intimate partner violence, a total of 86 (58.1%) experienced at least one form of IPV between baseline and endline surveys. A total of 133 (89.9%) women remained adherent to all four COVID-19 prevention strategies between baseline and endline surveys as shown in Table 1.

**Table 1:** Baseline characteristics of women living in informal settlements of Kampala, N = 148.

	Mean	SD
**Age, years**	32.9	9.3
	**Freq**	**Percent**
**Age category, years**		
18–24	28	18.9
25–34	56	37.8
35–44	48	32.4
45–64	16	10.8
**Education level**		
Secondary+	53	35.8
Primary	80	54.1
None	15	10.1
**Marital status**		
Single	19	12.8
Married/cohabiting	75	50.7
Divorced/widow	54	36.5
**Household size**		
1	2	1.4
2 to 4	81	54.7
5+	65	43.9
**Occupation**		
Employed/Self employed	107	72.3
Unemployed/student	41	27.7
**Problem getting food**		
Yes	115	78.2
No	32	21.8
**Intimate partner violence**		
No	62	41.9
Yes	86	58.1
Adherence to COVID-19 preventive measures*		
Poorly adherent	15	10.1
Remained adherent	133	89.9

*Computed as adherence between baseline and endline surveys

### Unadjusted and adjusted analysis

In the unadjusted logistic regression model, women who remained adherent to all four COVID-19 preventive measures had three times higher odds of experiencing atleast one form of IPV when compared to women who were poorly adherent to all four COVID-19 preventive guidelines [Odds Ratio 95% Confidence Interval 3.00 (1.04, 8.69)] as shown in Table 2.

**Table 2:** Unadjusted and adjusted logistic regression models for the independent association between adherence to COVID-19 preventive measures and intimate partner violence.

	Outcome: Intimate Partner Violence			
	Unadjusted models		Adjusted model	
	OR (95% CI)	p-value	OR (95% CI)	p-value
**Adherence to COVID-19 preventive measures**				
Poorly adherent	1		1	
Remained adherent	**3.00 (1.04, 8.69)**	**0.043**	**3.87 (1.09, 13.79)**	**0.037**
Age, years				
18–24	1		1	
25–34	0.93 (0.58, 1.50)	0.764	0.92 (0.28, 3.08)	0.897
35–44	0.73 (0.52, 1.02)	0.068	0.62 (0.17, 2.22)	0.465
45–64	1.33 (0.71, 2.50)	0.370	1.29 (0.19, 8.81)	0.794
Formal education				
Secondary+	1		1	
Primary	0.92 (0.37, 2.26)	0.849	1.03 (0.29, 3.69)	0.965
None	0.47 (0.26, 0.88)	0.018	0.45 (0.25, 0.84)	0.012
Marital status				
Single	1		1	
Married/cohab	1.37 (0.80, 2.33)	0.253	1.40 (0.63, 3.14)	0.408
Divorced/widow	1.87 (0.79, 4.41)	0.155	1.94 (0.75, 4.98)	0.171
Household size				
One	1		1	
2 to 4	0.80 (0.61, 1.06)	0.115	0.77 (0.32, 1.86)	0.565
5+	0.55 (0.49, 0.62)	<0.001	0.57 (0.24, 1.37)	0.208
Occupation				
(Self)employed	1		1	
Unemployed/student	0.59 (0.32, 1.10)	0.097	0.88 (0.57, 1.37)	0.565
Problem getting food				
No	1		1	
Yes	2.04 (1.18, 3.51)	0.011	2.02 (1.26, 3.25)	0.003

In the adjusted logistic regression model (Table 2), after controlling for potential confounders, women who remained adherent to all four COVID-19 preventive measures was independently associated with 3.87 times higher odds of experiencing at least one form of IPV when compared to women who were poorly adherent to all four COVID-19 preventive guidelines (OR 3.87, 95%CI 1.09, 13.79).

## Discussion

This study examined the association between adherence to COVID-19 preventive measures and intimate partner violence among women living in informal settings of Kampala during the first wave of COVID-19 in Uganda. Our results supported our hypothesis showing that IPV exposure differed significantly between women who remained adherent and women who were poorly adherent to preventive measures for COVID-19. We revealed three key findings. First, nearly half of the women surveyed in informal settlements experienced at least one form of IPV during the first COVID-19 wave in Uganda. Secondly, ten percent of women surveyed in informal settlements experienced were poorly adherent to all four COVID-19 preventive measures between our baseline and endline surveys. Thirdly, our analysis revealed that women in informal settings who remained adherent to COVID-19 preventive guidelines were more likely to experience intimate partner violence when compared to those who were poorly adherent.

Our study adds to the current body of knowledge on the relationship between IPV exposure and adherence to COVID-19 preventive measures among women in informal settings. First, our study described how women in informal settlements who remained adherent to all four preventive measures for COVID-19 over a six-week period during the first wave of COVID-19 were more likely to experience IPV when compared to their counterparts who were poorly adherent. Our findings were in line with previous research conducted in Kampala which showed that lower satisfaction in COVID-19 preventive measures was associated with experiencing violence and discrimination within homes perpetrated by family members [30]. The prior study was conducted among an online sample of women and men which suggests differences with current study. However, the prior study evaluated satisfaction to COVID-19 preventive measures using similar COVID-19 preventive measures as the current study.

Several factors may have contributed to the high burden of IPV associated with adherence to COVID-19 preventive measures among an already vulnerable population of women living in informal settlements of Kampala. Travel restrictions during the first COVID-19 wave prolonged exposure of women with potential perpetrators which could increase the risk of partner abuse. The context of implementation and compliance with COVID-19 preventive restrictions is important due to the economic effects on households such as loss of employment. Failure to obtain alternative income sources while adhering to ‘stay-at-home’ orders increases financial and psychological distress among bread winners. Job loss and hopelessness in the general community were common during the COVID-19 lockdown period in Uganda. Our study cohort were surveyed within a four month period between conformation of the incident COVID-19 case in Uganda on March 21, 2020 [40] and the first presidential directive for a total country lock down. This relatively lengthy and ongoing period of lockdowns and curfews may have been a major source of psychological and economic distress due to loss of household livelihoods hence translating into spousal abuse as a coping mechanism. Such stressful home environments compounded with an increase in household demands especially for supplies or bills raises the risk of abusive behavior as a compensatory mechanism. An imbalance in power dynamics in households during enforcement of COVID-19 restrictions may led to retaliatory abuse by men when the influence, decision making and capacity to provide financial support to their families was challenged. According to the theory of gender and power, IPV may have increased if men were rendered ‘powerless’ in a society that culturally condones male dominance. In addition, ‘stay-at-home’ directives may have aggravated perpetuation of IPV because gender roles that culturally assigned household provision roles to men challenged their masculinity hence men resorting to violence.

Our study also suggested that food insecurity may contribute to the high burden of IPV among women in informal settings. In fact, eighty percent of women in our study could not physically access or afford food reiterating prior research which showed the impact of COVID-19 on increased food insecurity [41]. This was in line with a population-based survey in rural and urban settings in Uganda that revealed an association between male partner perpetration of IPV and food insecurity [42]. It is plausible to suggest that a combination of stressors namely, economic (job loss poverty), and psychological (social isolation) could trigger domestic violence, especially among intimate partners. Several households lost their sources of income especially the men who are usually bread winners in households. This affected the livelihoods and quality of lives of women and their families especially those in disadvantaged communities such as informal settings.

Despite these potential reasons explaining why adherence to COVID-19 preventive measures may increase IPV risk, a need still exists to comprehensively examine the underlying factors for this increased IPV risk. Therefore, we posit that with gradual return to ‘normalcy’ in terms of easing COVID-19 restrictions and increasing economic activity, women impacted most by the financial distress and physical confinement, the duration of exposure to potential perpetrators and risk of IPV may reduce. Current efforts to achieve herd immunity with COVID-19 vaccinations and recently with the development of a COVID-19 pill [43] shows promise of likely easing of COVID-19 restrictions and potential reduction in IPV risk in households in informal settings.

Overall, present findings that reiterate the need for Uganda’s COVID-19 prevention and control measures to be inclusive, contextually tailored and responsive to the needs of marginalized groups. For example, our study was conducted among women dwelling in informal settlements who particularly lack safety nets to enable survival during such crises as the COVID-19 pandemic. Some government efforts aimed at addressing social needs in Uganda’s COVID-19 response included the provision of food relief to the urban poor and temporary suspension to closing some businesses due to non-tax payment. Other ad-hoc interventions to address the challenge of increasing IPV reports following presidential lockdown directives included law enforcement establishing a toll-free hotline with the Uganda Police Force to facilitate reporting of and response to IPV cases during and after the COVID-19 lockdown [44]. This study highlights the need to integrate violence prevention messages into the phased implementation of COVID-19 prevention interventions. This may increase awareness among informal settlement dwellers and a sense of urgency of the public health threat posed by COVID-19 transmission and acquisition. Therefore, government directives for mandatory face mask-wearing and restricting work to ‘essential’ workers-only may have driven the adoption of COVID-19 preventive measures over time. This is supported by the fact that facemask-wearing was acceptable as a preventive measure for COVID-19 particularly by urban dwellers in Kampala [45].

## Study implications

Regarding community practice, there is a need for relevant government ministries and stakeholders to engage in continuously implementing COVID-19 preventive measures while exploring underlying social needs and risks to for IPV among informal settlement dwellers that need to be addressed. Consistent community sensitization about the importance of COVID-19 preventive measures is required especially among the urban poor in Uganda. The national COVID-19 preventive task force should integrate IPV survivors who are experiencing prolonged exposure to IPV perpetrators by addressing their safety and psychosocial needs. For future research, although prior studies have conducted predictive models of COVID-19 on mortality, Disease Adjusted Life Years (DALYs), and the economic impact in Uganda, we recommend similar approaches to predict the long-term impact on COVID-19 preventive measures on IPV exposure as behavioral outcome during COVID-19 waves or similar outbreaks in Uganda.

The current policy discourse recommends equity in COVID-19 vaccination among most at-risk persons namely, healthcare workers, and elderly persons. There is a need to promote equity in the allocation of resources (food relief, vaccines, etc.) particularly to women who are marginalized, vulnerable and the urban poor. These populations include women living in informal settings because of their high psychosocial and economic needs and higher risk of exposure to IPV during the COVID-19 restriction period.

### Study strengths and limitations

This study was conducted in three divisions of Kampala therefore our findings are not representative of informal settings in all five divisions of Kampala but can be generalized to women dwelling in the informal settings studied in Kampala.

### Ethical considerations

Written informed consent was obtained from all study participants prior to each interview. Study participation was voluntary. Participants were assured of privacy, anonymity, and confidentiality during and after conducting interviews. Participants were free to withdraw from this study at any time without any consequences. There were no direct benefits or risks associated with participating in this study. As a risk mitigation strategy for COVID-19 transmission, data collectors wore face masks and maintained social distancing with each participant to reduce the risk of COVID-19 transmission during interviews. Ethical approval to conduct this study was obtained from the Higher Degrees and Research Ethics Committee (HDREC) of Makerere University School of Public Health and Uganda National Council of Science and Technology (UNCST) respectively.

## Conclusions

A substantial proportion of women dwelling in informal settlements of Kampala experienced at least one form of IPV during the first COVID-19 wave in Uganda. Exposure to IPV was more likely among women who remained adherent to COVID-19 preventive measures when compared to their counterparts who were poorly adherent to COVID-19 preventive measures between baseline and endline. Ongoing implementation of interventions to prevent transmission of COVID-19 should take the social needs and risks for IPV of marginalized and vulnerable women living in informal settings of Kampala into account. Integration of violence prevention and response strategies into the national COVID-19 prevention strategy for Uganda should be urgently initiated.

## Supporting information

S1 Data (DTA)
